# Path planning and obstacle avoidance with ISS framework for a UAV swarm under unified wind, sensor noise and delay disturbances

**DOI:** 10.1371/journal.pone.0352858

**Published:** 2026-07-14

**Authors:** Mehmet Karahan, Cosku Kasnakoglu

**Affiliations:** Department of Electrical and Electronics Engineering, TOBB University of Economics and Technology, Ankara, Turkiye; Zhejiang University, CHINA

## Abstract

**Background:**

Multi-UAV swarm systems have attracted significant attention in recent years due to their wide range of applications, including surveillance, disaster management, search and rescue, agriculture, infrastructure inspection, and autonomous transportation. In such systems, maintaining formation integrity, achieving accurate trajectory tracking, and ensuring safe obstacle avoidance under environmental and communication disturbances remain challenging research problems. Existing studies generally investigate wind effects, sensor noise, or communication delays separately and often lack a unified stability framework capable of characterizing their combined influence on formation performance.

**Objective:**

This study aims to develop a unified formation control and obstacle avoidance framework for multi-UAV systems operating under simultaneous wind disturbances, sensor noise, and communication delays. The proposed framework seeks to ensure stable formation regulation, centroid trajectory tracking, inter-agent collision prevention, and obstacle avoidance while providing formal robustness guarantees through an input-to-state stability (ISS) analysis.

**Methodology:**

A consensus-based Laplacian formation controller was integrated with centroid tracking and artificial-potential-field-based obstacle and collision avoidance mechanisms. The Crazyflie 2.0 Nano-Quadrotor model was employed, and the six-degree-of-freedom dynamics were simplified into planar motion for swarm-level analysis. Stability of the nominal system was investigated using Lyapunov theory, while the disturbed system was analyzed within an ISS framework to derive explicit steady-state tracking error bounds under bounded disturbances. Numerical simulations were conducted in MATLAB for different swarm formations and disturbance scenarios.

**Results:**

Simulation results demonstrated successful formation acquisition, centroid tracking, obstacle avoidance, and collision-free navigation under unified wind, sensor noise, and delay disturbances. Both hexagonal and line formations maintained stability while navigating toward desired target positions in the presence of static obstacles. Temporary formation deformations caused by avoidance maneuvers were effectively corrected, and the swarm recovered the desired geometry after disturbance effects diminished. Furthermore, the steady-state tracking errors remained within the theoretical ISS bounds, confirming consistency between the analytical results and simulation outcomes.

**Conclusion:**

The proposed framework provides a robust and unified solution for formation control and obstacle avoidance in disturbed multi-UAV systems. The integration of consensus-based control, artificial potential fields, and ISS-based robustness analysis enables reliable trajectory tracking and safe swarm coordination under realistic operating conditions. The obtained results indicate that the framework can serve as an effective foundation for future studies involving dynamic obstacles, three-dimensional swarm coordination, and real-world experimental validations.

## 1. Introduction

In recent years, studies on multi-UAV systems have become widespread. Such systems are utilized in many different areas, such as agriculture, combating natural disasters, surveillance, first aid, and entertainment [1,2]. Swarm drones are used to detect diseases by monitoring large agricultural lands for spraying, sowing seeds, and fertilizing [3,4]. They are employed to find missing people in natural disasters and to deliver first aid supplies [5,6]. Swarm drones are used to coordinate the delivery of emergency medical supplies, food, or communication equipment to isolated areas before ground rescue teams arrive [7]. Swarm UAVs can autonomously map and inspect bridges, buildings, pipelines, and wind turbines [8,9]. Swarm drones are also used to monitor faults, vegetation intrusion, or damage occurring along extensive power lines [10]. Swarm drones act as temporary, mobile base stations to provide network coverage in crowded or remote areas [11]. Multi-UAV systems are also utilized to track the spread of forest fires, assess damage, and support firefighting efforts [12,13]. Today, as an alternative to traditional, environmentally polluting fireworks, highly coordinated swarms of LED drones capable of creating 3D shapes and patterns are being used for entertainment and display purposes [14,15]. Swarm UAVs used for shows and entertainment cause less disturbance to humans and animals compared to fireworks. [16,17].

Numerous researchers are working on path planning and obstacle avoidance for swarm drones. In their recent studies, these researchers have primarily focused on repulsive potential field and artificial-potential-field-based path planning for swarm UAVs. Liu et al. addressed the limitations of conventional 2D drone navigation by introducing a 3D path-planning algorithm that models obstacle constraints in three-dimensional space for precise obstacle avoidance. They upgraded the traditional APF approach to account for and eliminate localized self-locking oscillations [18]. Zhen et al. proposed an intelligent cooperative mission planning scheme for a UAV swarm tasked with searching for and attacking time-sensitive moving targets within uncertain, dynamic environments. They combined a Hybrid Artificial Potential Field (HAPF) with a Distributed Ant Colony Optimization (ACO) algorithm [19]. Ruiz et al. addressed the growing need for real-time, autonomous trajectory planning for UAVs across various industries (surveillance, exploration, rescue, transport) by tackling a classic limitation of the Artificial Potential Field (APF) method: scaling attractive and repulsive forces. They introduced a novel 3D repulsive potential field built on fractional calculus to better scale navigation forces [20]. Goricanec et al. proposed an obstacle avoidance algorithm for UAVs navigating unknown environments while following a pre-planned trajectory. It is designed to work in real-time with 2D or 3D sensors, such as LiDAR. They upgraded the standard Artificial Potential Field approach by combining two attractive forces with both normal and rotational repulsive forces [21]. Zeng et al. proposed a multi-strategy flight planning method for UAVs designed to navigate obstacles safely while maintaining formation. They introduced a follow-up rotating vector field method specifically designed to navigate around convex polyhedral obstacles [22]. Antony et al. developed an approach for fixed wing UAV formation flying and obstacle avoidance while tracking a moving target. The proposed structure is maintained using attractive and repulsive artificial potential fields (APF) to track mobile targets with minimal error [23]. Rao et al. proposed a hybrid APF-A* (Artificial Potential Field – A*) algorithm designed for path planning in a dual-quadrotor cooperative heavy-load transport system [24]. Heidari et al. developed trajectory tracking and obstacle avoidance algorithms for UAVs under wind using a modified artificial potential field [25]. Yu et al. presented a refined fault tolerant tracking control (FTTC) scheme for multiple fixed-wing UAVs that addresses actuator faults and wind effects while reducing communication overhead [26]. Chen et al. presented an enhanced Artificial Potential Field (APF) method for path planning of Unmanned Surface Vehicles (USVs) in complex marine environments. Their research addressed key challenges including insufficient obstacle clearance, heading oscillations, and poor wind-current disturbance rejection, particularly in scale-constrained narrow channels [27].

Although the studies on multi-UAV systems explored coordination, obstacle avoidance, and robust control, several gaps still remain. Most existing studies examine disturbances such as wind, sensor noise, or communication delays in isolation, rather than within a unified framework that captures their combined effect. Moreover, stability analyses commonly demonstrate convergence under nominal conditions and do not provide explicit input-to-state characterizations that relate steady-state formation error to uncertainty bounds. These limitations motivate the present work.

In this study, we introduce a unified control approach that keeps a multi-UAV formation intact, enables the group to move toward a desired location, incorporates obstacle and collision avoidance, and offers formal stability guarantees under realistic disturbances. The controller is built around a Laplacian-based formation law supplemented with repulsive potentials for obstacle and inter-agent safety. An input-to-state stability (ISS) analysis is developed to characterize the steady-state behavior of the multi-UAV system when exposed to wind, measurement noise, and communication delay.

The main contributions of this study can be summarized as follows:

1) A unified and implementable control architecture that merges formation control, tracking, obstacle avoidance, and inter-UAV collision prevention.
2) A realistic disturbance model capturing wind, sensor noise, and delays.
3) An ISS stability analysis yielding bounds that relate disturbance strength to steady-state error.
4) Simulation results demonstrating formation acquisition, trajectory tracking, obstacle avoidance, collision prevention, and agreement between the errors and the ISS bound.

Section 1 gives the introduction. Section 2 explains the methodology. Section 3 presents the stability analysis. Section 4 provides the results. Finally, Section 5 summarizes the conclusions of the study.

## 2. Methodology

In this section, the quadrotor model used in the multi-UAV setup, formation creation, leader-follower approach, reaching the predetermined target and obstacle avoidance features are explained.

### 2.1. Quadrotor model

**Fig 1:**
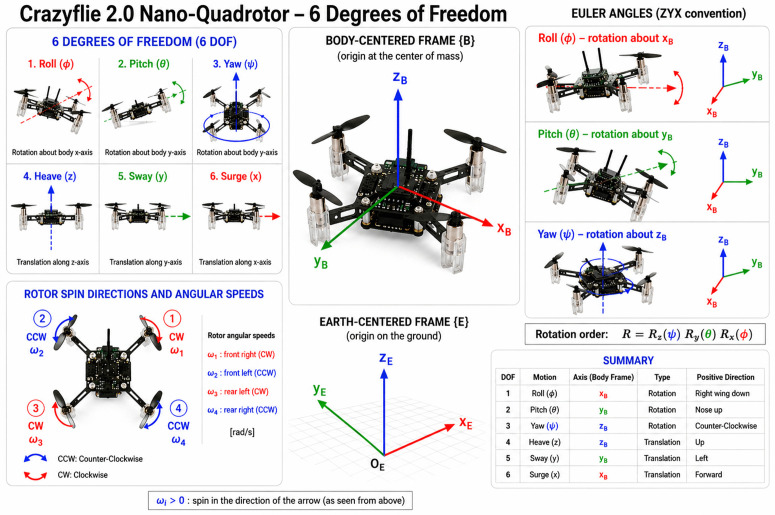
The Crazyflie 2.0 Nano-Quadrotor.

The quadrotor is a four-rotor UAV that performs VTOL and moves in six degrees of freedom (DOF). Quadrotor’s movements in the x, y, z axes and rotational movements in the roll (θ), pitch (ϕ), yaw (ψ) angles constitute 6 DOF [28]. The Crazyflie 2.0 Nano-Quadrotor model was used in this research. This quadrotor is suitable for use in multi-UAV studies due to its small size, light weight, and programmability. Fig 1 shows in detail the motion of the Crazyflie 2.0 Nano-Quadrotor used in this study at 6 DOF. The rotor rotation direction, angular velocities, body-axis frame, ground-axis frame, and Euler angles are also shown in Fig 1.

A quadrotor UAV operates with 6 DOF. To reduce this 6-degree-of-freedom motion to planar motion along the x-y axis, certain assumptions must be made. First, a constant altitude assumption is made.


$$\begin{array}{c} z=constant \end{array}$$ (1)

With a constant altitude assumption, the second derivative of z becomes 0.


$$\begin{array}{c} \ddot{z}=\text{0} \end{array}$$ (2)

In this case, the Torque value is obtained as in equation (3).


$$\begin{array}{c} T\approx mg \end{array}$$ (3)

With the small-angle approximation, the assumptions given in equation (4) are valid around the hover.


$$\begin{array}{c} \;\begin{array}{c} sin\phi \approx \phi \\ sin\theta \approx \theta \\ cos\phi \approx \text{1}\\ cos\theta \approx \text{1} \end{array}\;\;\} \end{array}$$ (4)

Assuming the yaw angle is close to 0, the following approximations are valid.


$$\begin{array}{c} \psi \approx \text{0} \end{array}$$ (5)

In this case, the results in equation (6) are obtained.


$$\begin{array}{c} \;\begin{array}{c} cos\psi \approx \text{1}\\ sin\psi \approx \text{0} \end{array}\;\;\;\} \end{array}$$ (6)

As a result of these simplifications, the X-Y dynamics are obtained as in equation (7).


$$\begin{array}{c} \;\begin{array}{c} \ddot{x}\approx g\theta \\ \ddot{y}\approx -g\phi \end{array}\;\;\} \end{array}$$ (7)

According to this result, the pitch angle (θ) produces forward/backward motion, and the roll angle (ϕ) produces right/left motion.

In a system with 2 degrees of freedom, the state vector is as in equation (8).


$$\begin{array}{c} {x=[x,\dot{x},y,\dot{y}]}^{T} \end{array}$$ (8)

The system input is as in equation (9).


$$\begin{array}{c} {u=[\theta ,\phi ]}^{T} \end{array}$$ (9)

The state space and the vectors A and B of the state space are given in equations (10)-(12).


$$\begin{array}{c} \dot{x}=Ax+Bu \end{array}$$ (10)

$$\begin{array}{c} A=[\begin{array}{cccc} \text{0} & \text{1} & \text{0} & \text{0}\\ \text{0} & \text{0} & \text{0} & \text{0}\\ \text{0} & \text{0} & \text{0} & \text{1}\\ \text{0} & \text{0} & \text{0} & \text{0} \end{array}] \end{array}$$ (11)

$$\begin{array}{c} B=[\begin{array}{cc} \text{0} & \text{0}\\ g & \text{0}\\ \text{0} & \text{0}\\ \text{0} & -g \end{array}] \end{array}$$ (12)

**Fig 2:**
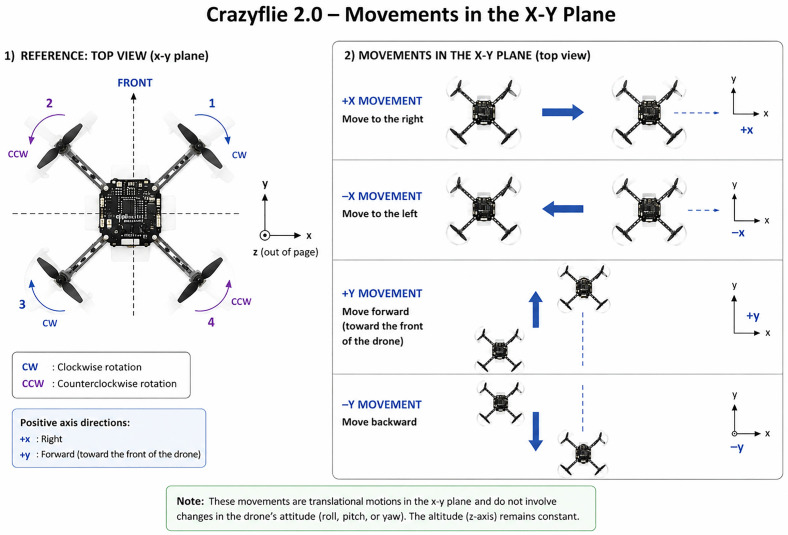
Two-dimensional movements of the Crazyflie 2.0 Nano-Quadrotor in the X-Y plane.

A schematic showing the two-dimensional movements of the Crazyflie 2.0 Nano-Quadrotor in the X-Y plane is given in Fig 2.

Technical information about the employed quadrotor is given in Table 1 [29,30].

**Table 1:** Crazyflie 2.0 Nano-Quadrotor Parameters.

Parameter	Value
mass (m)	33 g
arm length (l)	39.73 x 10^−3^ m
inertial moment on x-axis (I_x_)	1.395 x 10^−5^ kgm^2^
inertial moment on y-axis (I_y_)	1.436 x 10^−5^ kgm^2^
inertial moment on z-axis (I_z_)	2.173 x 10^−5^ kgm^2^
inertia of rotor (J_r_)	0 kgm^2^
maximum rotor speed (w_max_)	3000 rad/s
motor constant (k_F_)	2.8799 x 10^−8^
moment constant (k_M_)	7.2385 x 10^−10^
onboard microcontroller	STM32F405 main application MCU
gyro	3-axis gyro (MPU-9250)
accelerometer	3-axis accelerometer (MPU-9250)
magnetometer	3-axis magnetometer (MPU-9250)
pressure sensor	high precision pressure sensor (LPS25H)
flight time	7 minutes
charging time	40 minutes
maximum payload weight	15 g

### 2.2. Formation shape regulation

To maintain the formation, each UAV is assigned an ideal offset in the final formation. These are points on the corners of the shape. As an example, we use a regular hexagon for our simulations, but the method works for any shape so long as the offsets are defined properly.

Each UAV looks at its neighbors and compares their relative positions to the ones they should have in the formation. A control action is generated based on the errors in these as follows


$$\begin{array}{c} \overset{\text{form}}{\underset{i}{v}}=k_{p}\sum_{j=\text{1}}^{N}a_{ij}\left[\left(p_{j}-p_{i}\right)-\left(f_{j}-f_{i}\right)\right].\; \end{array}$$ (13)

Where N is the UAV count, $p_{i}\in R^{\text{3}}$ is the position of UAV i, $f_{i}\in R^{\text{3}}$ is the desired formation offset assigned to UAV i, $k_{p}>\text{0}\;$is the formation tracking gain, $a_{ij}$ is the adjacency weight describing whether UAV i uses the state of UAV j in its formation control law or not. $\left(p_{j}-p_{i}\right)-\left(f_{j}-f_{i}\right)$ is therefore the relative formation error between UAV i and j. The control action can be expressed in Laplacian form as


$$\begin{array}{c} \overset{\text{form}}{\underset{i}{v}}=-k_{p}\sum_{j=\text{1}}^{N}\ell_{ij}\;\left(p_{j}-f_{j}\right) \end{array}$$ (14)

where ${\text{𝓁}}_{ij}$ is an element of the graph Laplacian $𝐋=\left[{\text{𝓁}}_{ij}\right]=𝐃-𝐀$. Here $𝐀=\left[a_{ij}\right]$ is the adjacency matrix and $𝐃=\text{diag}\left(d_{\text{1}},\ldots ,d_{N}\right)$ is the degree matrix with $d_{i}=\sum_{j=\text{1}}^{N}a_{ij}$. For all-to-all communication topology considered in this work we have


$$\begin{array}{c} \begin{array}{c} d_{i}=N-\text{1} \end{array} \end{array}$$ (15)

and


$$\begin{array}{c} \begin{array}{c} a_{ij}=\left\{ \begin{array}{cc} \text{1}, & i\text{≠}j,\\ \text{0}, & i=j \end{array}\right.\; \end{array} \end{array}$$ (16)

which imply 𝐃=(N−1)𝐈 and $𝐀=11^{T}-𝐈$ so


$$\begin{array}{c} \begin{array}{c} 𝐋=(N-\text{1})𝐈-\left(11^{T}-𝐈\right)=N𝐈-11^{T}. \end{array} \end{array}$$ (17)

### 2.3. Centroid tracking

The motion of the multi-UAV team’s centroid to track a desired trajectory is achieved by the control action


$$\begin{array}{c} \begin{array}{c} \overset{\text{cent}}{\underset{i}{v}}=-k_{c}\left(c-c_{d}\right)+{\dot{c}}_{d}, \end{array} \end{array}$$ (18)

where $c=\frac{\text{1}}{N}\sum_{j=\text{1}}^{N}p_{j}$ is the team’s centroid and $c_{d}(t)$ is the desired trajectory. This same term is added to all UAVs, making the entire group shift in the desired direction.

### 2.4. Obstacle and collision avoidance

During the flight, the formation must avoid environmental obstacles. When quadrotor-i senses an obstacle $o_{h}$, it will produce a repulsive action as below to avoid it. In the equations below, r_s_ represents the detection distance between a UAV and an obstacle. r_min_ represents the minimum distance between a UAV and an obstacle. d_safe_ represents the safe distance between two UAVs. d_min_ represents the minimum distance between two UAVs. The minimum distance is calculated as half of the safe distance.


$$\begin{array}{c} \begin{array}{c} \overset{\text{obs}}{\underset{i,h}{v}}=\left\{ \begin{array}{cc} k_{\text{0}}\left(\frac{\text{1}}{\overset{\text{2}}{\underset{\text{min}}{r}}}-\frac{\text{1}}{\overset{\text{2}}{\underset{s}{r}}}\right)\frac{p_{i}-p_{oh}}{\|p_{i}\;-\;p_{oh}\|},\; & {\;d}_{ih}\leq r_{\text{min}}\\ k_{\text{0}}\left(\frac{\text{1}}{\overset{\text{2}}{\underset{ih}{d}}}-\frac{\text{1}}{\overset{\text{2}}{\underset{s}{r}}}\right)\frac{p_{i}-p_{oh}}{\|p_{i}\;-\;p_{oh}\|},\; & {{\;r}_{\text{min}}<d}_{ih}\leq r_{s}\\ \text{0},\; & {\;d}_{ih}>r_{s} \end{array}\right.\; \end{array} \end{array}$$ (19)

where $k_{o}\;>\;\text{0}$ is the obstacle repulsion gain, $r_{s}$ is the sensing distance, $r_{\text{min}}$ is the minimum admissible distance between any UAV and any obstacle, $p_{i}$ is the position of the quadrotor, $p_{oh}$ is the center of obstacle $o_{h}$, and $d_{ih}=\|p_{i}\;-\;p_{oh}\|$ is the distance between the quadrotor-i and obstacle $o_{h}$. When all obstacles are taken into account, the obstacle avoidance behavior of UAV-i is as below


$$\begin{array}{c} \overset{\text{obs}}{\underset{i}{v}}=\sum_{h=\text{1}}^{M}\overset{\text{obs}}{\underset{i,h}{v}} \end{array}$$ (20)

where M is the number of obstacles.

UAVs within the formation must also avoid colliding with one another. If UAV-i senses UAV-j closer than a safe distance, a repulsive action


$$\begin{array}{c} \begin{array}{c} \overset{\text{coll}}{\underset{i,j}{v}}=\left\{ \begin{array}{cc} k_{\text{coll}}\left(\frac{\text{1}}{\overset{\text{2}}{\underset{\text{min}}{d}}}-\frac{\text{1}}{\overset{\text{2}}{\underset{s}{r}}}\right)\frac{p_{i}-p_{j}}{∥p_{i}-p_{j}∥},\; & {\;d}_{ij}\leq d_{\text{min}}\\ {\;k}_{\text{coll}}\left(\frac{\text{1}}{\overset{\text{2}}{\underset{ij}{d}}}-\frac{\text{1}}{\overset{\text{2}}{\underset{\text{safe}}{d}}}\right)\frac{p_{i}-p_{j}}{∥p_{i}-p_{j}∥},\; & {\;d_{\text{min}}<d}_{ij}\leq d_{\text{safe}}\\ \text{0},\; & {\;d}_{ij}>d_{\text{safe}} \end{array}\right.\; \end{array} \end{array}$$ (21)

will be applied to UAV-i. Here $k_{\text{coll}}>\text{0}$ is the inter-UAV repulsion gain, $d_{\text{safe}}$ is the safe distance, $d_{\text{min}}$ is the minimum admissible distance between any two UAVs, $p_{i}$ is the position of UAV-i, $p_{j}$ is the position of UAV-j, $d_{ij}=∥p_{i}-p_{j}∥$ is the distance between UAVs i and j. When all UAVs are considered, the collision avoidance behavior of UAV-i is as follows


$$\begin{array}{c} \overset{\text{coll}}{\underset{i}{v}}=\sum_{j=\text{1}}^{N}\overset{\text{coll}}{\underset{i,j}{v}} \end{array}$$ (22)

where N is the number of UAVs.

### 2.5. Disturbances

In this work we consider three types of disturbances. Wind forcing $\overset{\text{wind}}{\underset{i}{w}}(t)\in R^{\text{3}}$ is modeled as a random bounded disturbance added to the UAV speed. Measurement noise $\eta_{i}(t)\in R^{\text{3}}$ and communication delay $\tau_{ij}(t)$ are bounded random quantities affecting position measurements as


$$\begin{array}{c} \begin{array}{c} \overset{\text{meas}}{\underset{i}{p}}(t)=p_{i}\left(t-\tau_{ij}(t)\right)+\eta_{i}(t) \end{array} \end{array}$$ (23)

Since all disturbances are assumed bounded, for analysis we shall treat them as bounded exogenous terms added to the control action and lump them into a single term as


$$\begin{array}{c} \begin{array}{c} \overset{\text{exo}}{\underset{i}{w}}(t)=\overset{\text{wind}}{\underset{i}{w}}(t)+\overset{\text{noise}}{\underset{i}{w}}(t)+\overset{\text{delay}}{\underset{i}{w}}(t) \end{array} \end{array}$$ (24)

with bound $∥\overset{\text{exo}}{\underset{i}{w}}(t)∥\leq \overset{\text{exo}}{\underset{i}{\overset{―}{w}}}$.

## 3. Stability analysis

This section presents the system model and performs a stability analysis using mathematical theorems.

### 3.1. System model

The dynamics of UAV-i in the formation can be modeled as


$$\begin{array}{c} \begin{array}{c} {\dot{p}}_{i}=u_{i}+w_{i},\;i=\text{1},\text{2},\ldots ,N \end{array} \end{array}$$ (25)

where $p_{i}(\text{t})={\left[x_{i}(t),y_{i}(t),z_{i}(t)\right]}^{T}\in R^{\text{3}}$ denotes the UAV position, $u_{i}(t)\in R^{\text{3}}$ is the control input


$$\begin{array}{c} \begin{array}{c} u_{i}=\overset{\text{form}}{\underset{i}{v}}+\overset{\text{cent}}{\underset{i}{v}} \end{array} \end{array}$$ (26)

and


$$\begin{array}{c} \begin{array}{c} w_{i}(t)\;=\;\overset{\text{obs}}{\underset{i}{v}}(t)+\overset{\text{coll}}{\underset{i}{v}}(t)+\overset{\text{exo}}{\underset{i}{w}}(t),\;\;∥w_{i}(t)∥\leq {\bar{w}}_{i}. \end{array} \end{array}$$ (27)

That is, for purposes of stability analysis, we shall lump obstacle/collision avoidance forces as well as the wind, sensor noise, and delays effects into a single bounded disturbance $w_{i}(t)$.

The control input has the form


$$\begin{array}{c} u_{i}=k_{p}\sum_{j=\text{1}}^{N}\left(p_{j}-p_{i}-\left(f_{j}-f_{i}\right)\right)-k_{c}\left(\frac{\text{1}}{N}\sum_{j=\text{1}}^{N}p_{j}-c_{d}\right)+{\dot{c}}_{d} \end{array}$$ (28)

where $f_{i}$ is the desired geometric offset of ith UAV in the formation, $k_{p}>\text{0}$ and $k_{c}>\text{0}$ are control gains for formation and centroid tracking, and $c_{d}$ is the desired centroid trajectory.

Defining stacked position and desired formation vectors


$$\begin{array}{c} \begin{array}{c} 𝐩={\left[\overset{T}{\underset{\text{1}}{p}},\overset{T}{\underset{\text{2}}{p}},\ldots ,\overset{T}{\underset{N}{p}}\right]}^{T},\;𝐟={\left[\overset{T}{\underset{\text{1}}{f}},\overset{T}{\underset{\text{2}}{f}},\ldots ,\overset{T}{\underset{N}{f}}\right]}^{T} \end{array} \end{array}$$ (29)

the collective dynamics can be expressed as


$$\begin{array}{c} \begin{array}{c} \dot{𝐩}=-k_{p}\left(𝐋⊗𝐈\right)\left(𝐩-𝐟\right)-k_{c}\left(𝐌⊗𝐈\right)\left(𝐩-1⊗c_{d}\right)+1⊗{\dot{c}}_{d}+𝐰. \end{array} \end{array}$$ (30)

where $𝐋=N𝐈-11^{\text{T}}$ is the Laplacian matrix for an all-to-all communication graph, $𝐌=\frac{\text{1}}{N}11^{\text{T}}$ is the centroid averaging matrix, ⊗ denotes the Kronecker product and $𝐰={\left[\overset{T}{\underset{\text{1}}{w}},\ldots ,\overset{T}{\underset{N}{w}}\right]}^{T}$ is the disturbance vector.

Define the error dynamics


$$\begin{array}{c} \begin{array}{c} \delta =𝐩-\left(𝐟+1⊗c_{d}\right)=𝐩-𝐟-1⊗c_{d}\;. \end{array} \end{array}$$ (31)

which captures both the formation regulation and centroid tracking objectives. Differentiating gives


$$\begin{array}{c} \begin{array}{c} \dot{\delta }=\dot{𝐩}-1⊗{\dot{c}}_{d} \end{array} \end{array}$$ (32)

since $\dot{𝐟}=\text{0}$ because formation offsets are constant. Substituting $\dot{𝐩}$ from above


$$\begin{array}{c} \begin{array}{c} \dot{\delta }=-k_{p}\left(𝐋⊗𝐈\right)\left(𝐩-𝐟\right)-k_{c}\left(𝐌⊗𝐈\right)\left(𝐩-1⊗c_{d}\right)+𝐰 \end{array} \end{array}$$ (33)

For the first term note that $𝐩-𝐟=\delta +1⊗c_{d}$ but since $\left(𝐋⊗𝐈\right)\left(1⊗c_{d}\right)=\left(𝐋1\right)⊗c_{d}=\text{0}$ it follows (𝐋⊗𝐈)(𝐩−𝐟)=(𝐋⊗𝐈)δ. For the second term note $𝐩-1⊗c_{d}=\delta -𝐟$ but if we assume the formation offsets are centered, i.e., $\sum_{i}f_{i}=\text{0}$ then (𝐌⊗𝐈)𝐟=0 so it follows that $\left(𝐌⊗𝐈\right)\left(𝐩-1⊗c_{d}\right)=\left(𝐌⊗𝐈\right)\delta$. As a result


$$\begin{array}{c} \begin{array}{c} \dot{\delta }=-k_{p}\left(𝐋⊗𝐈\right)\delta -k_{c}\left(𝐌⊗𝐈\right)\delta +𝐰 \end{array} \end{array}$$ (34)

### 3.2. Related theorems

This section discusses the theorems and their explanations used in this study.

**Theorem 1 (Global Exponential Stability for No Disturbance Case)**: *For the nominal system* (w=0), *the equilibrium point* δ=0 *is globally exponentially stable for all* $k_{p},k_{c}>0$.

**Proof of Theorem 1:** Consider the Lyapunov function


$$\begin{array}{c} \begin{array}{c} V\left(\delta \right)=\frac{\text{1}}{\text{2}}∥\delta {∥}^{\text{2}}=\frac{\text{1}}{\text{2}}\delta^{T}\delta \end{array} \end{array}$$ (35)

Taking its derivative along system trajectories


$$\begin{array}{c} \begin{array}{c} \dot{V}=\delta^{T}\dot{\delta }=-k_{p}\delta^{T}\left(𝐋⊗𝐈\right)\delta -k_{c}\delta^{T}\left(𝐌⊗𝐈\right)\delta \end{array} \end{array}$$ (36)

Define the centroid (average) error $\delta_{\text{avg}}:=\frac{\text{1}}{N}\sum_{i=\text{1}}^{N}\delta_{i}$, the stacked average vector $\left(1⊗𝐈\right)\delta_{\text{avg}},\;$and the disagreement (shape) component as $\delta_{⟂}:=\delta -\left(1⊗𝐈\right)\delta_{\text{avg}}$. Then the error is decomposed into two orthogonal components as


$$\begin{array}{c} \begin{array}{c} \delta =\delta_{⟂}+\left(1⊗𝐈\right)\delta_{\text{avg}} \end{array} \end{array}$$ (37)

Note also $\sum_{i=\text{1}}^{N}{\left(e_{⟂}\right)}_{i}=\text{0}$ and that $∥\delta {∥}^{\text{2}}=∥\delta_{⟂}{∥}^{\text{2}}+N∥\delta_{\text{avg}}{∥}^{\text{2}}.\;$For the first term in $\dot{V}$ we have


$$\begin{array}{c} \begin{array}{c} \delta^{⊤}\left(𝐋⊗𝐈\right)\delta =\overset{⊤}{\underset{⟂}{\delta }}\left(𝐋⊗𝐈\right)\delta_{⟂}\geq \lambda_{\text{2}}∥\delta_{⟂}{∥}^{\text{2}} \end{array} \end{array}$$ (38)

which follows from (𝐋⊗𝐈)(1⊗𝐈)=(𝐋1)⊗𝐈=0 with $\lambda_{\text{2}}=\lambda_{\text{2}}\left(𝐋\right)$ being the smallest positive eigenvalue of 𝐋. For the second term in $\dot{V}$ we have


$$\begin{array}{c} \begin{array}{c} \delta^{⊤}\left(𝐌⊗𝐈\right)\delta ={\left(1⊗\delta_{\text{avg}}\right)}^{⊤}\left(𝐌⊗𝐈\right)\left(1⊗\delta_{\text{avg}}\right)=N∥\delta_{\text{avg}}{∥}^{\text{2}} \end{array} \end{array}$$ (39)

which follows from$\;\left(𝐌⊗𝐈\right)\delta_{⟂}=\text{0}$ and $𝐌=\frac{\text{1}}{N}11^{\text{T}}$ being the averaging matrix.

Using these results in the $\dot{V}$ expression gives


$$\begin{array}{c} \begin{array}{c} \dot{V}\leq -k_{p}\lambda_{\text{2}}∥\delta_{⟂}{∥}^{\text{2}}-k_{c}N∥\delta_{\text{avg}}{∥}^{\text{2}}\;\leq -\alpha_{\text{0}}\left(∥\delta_{⟂}{∥}^{\text{2}}+∥\delta_{\text{avg}}{∥}^{\text{2}}\right) \end{array} \end{array}$$ (40)

where $\alpha_{\text{0}}:=\text{min}\left\{\;k_{p}\lambda_{\text{2}},\;k_{c}N\;\right\}$ and $\lambda_{\text{2}}=N$ for an all-to-all graph. Using $∥\delta {∥}^{\text{2}}=∥\delta_{⟂}{∥}^{\text{2}}+N∥\delta_{\text{avg}}{∥}^{\text{2}}\;\leq N\left(∥\delta_{⟂}{∥}^{\text{2}}+∥\delta_{\text{avg}}{∥}^{\text{2}}\right)$ one obtains so


$$\begin{array}{c} \begin{array}{c} \dot{V}\leq -\alpha_{\text{0}}\left(∥\delta_{⟂}{∥}^{\text{2}}+∥\delta_{\text{avg}}{∥}^{\text{2}}\right)\leq -\frac{\alpha_{\text{0}}}{N}∥\delta {∥}^{\text{2}}=-\text{2}\left(\frac{\alpha_{\text{0}}}{N}\right)V \end{array} \end{array}$$ (41)

since $V=\frac{\text{1}}{\text{2}}∥\delta {∥}^{\text{2}}$. Hence


$$\begin{array}{c} \begin{array}{c} V(t)\leq V(\text{0})e^{-\text{2}{(\alpha }_{\text{0}}/N)t}\;\Rightarrow \;∥\delta (t)∥\leq ∥\delta (\text{0})∥e^{-{(\alpha }_{\text{0}}/N)t} \end{array} \end{array}$$ (42)

meaning that the error converges exponentially to zero.∎

**Theorem 2 (Input-to-State Stability (ISS) Under Disturbances)**: *Consider the disturbed system*


$$\begin{array}{c} \begin{array}{c} \dot{\delta }=-k_{p}\left(𝐋⊗𝐈\right)\delta -k_{c}\left(𝐌⊗𝐈\right)\delta +𝐰 \end{array} \end{array}$$ (43)

Assume 𝐰(t) is bounded, i.e., $∥𝐰(t)∥\leq \bar{w}$. Then the closed-loop system is input-to-state stable (ISS).

**Proof of Theorem 2:** Using the same Lyapunov function $=\frac{\text{1}}{\text{2}}∥\delta {∥}^{\text{2}}$ as in Theorem 1


$$\begin{array}{c} \begin{array}{c} \dot{V}\leq -\text{2}\frac{\alpha_{\text{0}}}{N}V+\delta^{T}𝐰 \end{array} \end{array}$$ (44)

Using Cauchy–Schwarz and Young inequalities


$$\begin{array}{c} \begin{array}{c} \delta^{T}𝐰\leq ∥\delta ∥\;∥𝐰∥\leq \frac{\epsilon }{\text{2}}∥\delta {∥}^{\text{2}}+\frac{\text{1}}{\text{2}\epsilon }∥𝐰{∥}^{\text{2}}\leq \epsilon V+\frac{\text{1}}{\text{2}\epsilon }∥𝐰{∥}^{\text{2}} \end{array} \end{array}$$ (45)

holds for any ϵ>0. Substituting this in the $\dot{V}$ expression


$$\begin{array}{c} \begin{array}{c} \dot{V}\leq -\left(\text{2}\frac{\alpha_{\text{0}}}{N}-\epsilon \right)V+\frac{\text{1}}{\text{2}\epsilon }∥𝐰{∥}^{\text{2}} \end{array} \end{array}$$ (46)

Choosing $\text{0}<\epsilon <\text{2}\frac{\alpha_{\text{0}}}{N}$ gives the ISS condition


$$\begin{array}{c} \begin{array}{c} V(t)\leq V(\text{0})e^{-c_{\text{1}}t}+\frac{c_{\text{2}}}{c_{\text{1}}}\bar{w} \end{array} \end{array}$$ (47)

where $c_{\text{1}}=\text{2}\frac{\alpha_{\text{0}}}{N}-\epsilon$ and $c_{\text{2}}=\frac{\text{1}}{\text{2}\epsilon }$.

Thus


$$\begin{array}{c} \begin{array}{c} ∥\delta (t)∥\leq ∥\delta (\text{0})∥e^{-\frac{c_{\text{1}}t}{\text{2}}}+\sqrt{\frac{\text{2}c_{\text{2}}}{c_{\text{1}}}}\bar{w} \end{array} \end{array}$$ (48)

which confirms ISS with a linear gain $\gamma (r)=\sqrt{\frac{\text{2}c_{\text{2}}}{c_{\text{1}}}}r=\sqrt{\text{1}/\left[\epsilon \left(\text{2}\alpha_{\text{0}}/N-\epsilon \right)\right]}\;r$. ∎

**Remark:** If we choose $\epsilon =\;\frac{\alpha_{\text{0}}}{N}\;$for example, then $c_{\text{1}}=\frac{\alpha_{\text{0}}}{N}$ and $c_{\text{2}}=\frac{N}{\text{2}\alpha_{\text{0}}}$ so $\gamma (r)=N/\alpha_{\text{0}}r$. Thus as t→∞, the error will be bounded with


$$\begin{array}{c} \begin{array}{c} {\text{lim sup}}_{t\to \infty }∥\delta (t)∥\leq \frac{\text{N}}{\alpha_{\text{0}}\bar{w}} \end{array} \end{array}$$ (49)

where $\overset{―}{w}$ is the disturbance bound, $\alpha_{\text{0}}:=\text{min}\left\{\;k_{p}\lambda_{\text{2}},\;k_{c}N\;\right\}$ and $\lambda_{\text{2}}=N$ for an all-to-all graph as considered in this work.

**Theorem 3 (Boundedness of Disturbance Components)**: The obstacle avoidance and collision avoidance terms are bounded, hence the total disturbance term satisfies the boundedness assumption in Theorem 2.

**Proof of Theorem 3:** Consider UAV-i. From (7) and (9) it is clear that the obstacle avoidance term $\overset{\text{obs}}{\underset{i,h}{v}}$ is bounded as


$$\begin{array}{c} \begin{array}{c} ∥\overset{\text{obs}}{\underset{i,h}{v}}∥\leq k_{\text{0}}\left(\frac{\text{1}}{\overset{\text{2}}{\underset{\text{min}}{r}}}-\frac{\text{1}}{\overset{\text{2}}{\underset{s}{r}}}\right) \end{array} \end{array}$$ (50)

and the inter-agent repulsion term $\overset{\text{coll}}{\underset{i,j}{v}}$ is bounded as


$$\begin{array}{c} \begin{array}{c} ∥\overset{\text{coll}}{\underset{i,j}{v}}∥\leq k_{\text{coll}}\left(\frac{\text{1}}{\overset{\text{2}}{\underset{\text{min}}{d}}}-\frac{\text{1}}{\overset{\text{2}}{\underset{\text{safe}}{d}}}\right)\;. \end{array} \end{array}$$ (51)

For a total of M obstacles and N UAVs, we have $\overset{\text{obs}}{\underset{i}{v}}=\sum_{h=\text{1}}^{M}\overset{\text{obs}}{\underset{i,h}{v}}$ and $\overset{\text{coll}}{\underset{i}{v}}=\sum_{j=\text{1}}^{N}\overset{\text{coll}}{\underset{i,j}{v}}$ (for i≠j) so


$$\begin{array}{c} \begin{array}{c} ∥\overset{\text{obs}}{\underset{i}{v}}∥+∥\overset{\text{coll}}{\underset{i}{v}}∥\;\leq \;{Mk}_{\text{0}}\left(\frac{\text{1}}{\overset{\text{2}}{\underset{\text{min}}{r}}}-\frac{\text{1}}{\overset{\text{2}}{\underset{s}{r}}}\right)+(N-\text{1})k_{\text{coll}}\left(\frac{\text{1}}{\overset{\text{2}}{\underset{\text{min}}{d}}}-\frac{\text{1}}{\overset{\text{2}}{\underset{\text{safe}}{d}}}\right) \end{array} \end{array}$$ (52)

Since we also have $∥\overset{𝐞𝐱𝐨}{\underset{i}{w}}∥\leq {\bar{w}}_{\text{exo}}$ from earlier, for the total disturbance norm for all N UAVs one can write


$$\begin{array}{c} \begin{array}{c} ∥𝐰∥\leq ∥{𝐰}^{𝐞𝐱𝐨}∥+∥{𝐯}^{𝐨𝐛𝐬}∥+∥{𝐯}^{𝐜𝐨𝐥}∥\leq \overset{―}{w} \end{array} \end{array}$$ (53)

where


$$\begin{array}{c} \begin{array}{c} \overset{―}{w}=N{\bar{w}}_{\text{exo}}+{NMk}_{\text{0}}\left(\frac{\text{1}}{\overset{\text{2}}{\underset{\text{min}}{r}}}-\frac{\text{1}}{\overset{\text{2}}{\underset{s}{r}}}\right)+N(N-\text{1})k_{\text{coll}}\left(\frac{\text{1}}{\overset{\text{2}}{\underset{\text{min}}{d}}}-\frac{\text{1}}{\overset{\text{2}}{\underset{\text{safe}}{d}}}\right) \end{array} \end{array}$$ (54)

is the total disturbance bound as assumed in Theorem 2.∎

**Fig 3:**
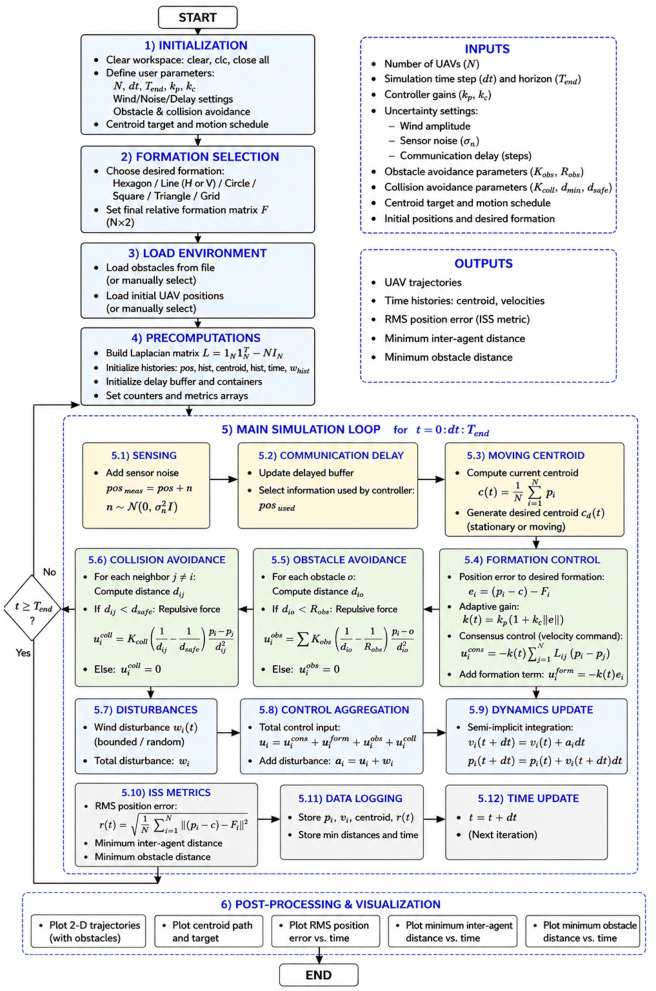
Algorithmic flowchart of the study.

**Remark:** The theoretical bound above is a worst-case upper bound and scales with the terms NM and N(N−1). This may produce overly conservative estimates, especially for large teams and many obstacles. In practice, obstacle and collision avoidance forces are not always active for all UAVs simultaneously; these forces are typically localized in time and space. To obtain a tighter estimate one may compute an empirical disturbance bound instead, e.g., by evaluating the disturbance value along the system trajectory and taking its maximum over time, which will be the path taken in the simulations to follow as well. The algorithmic flowchart of the study is given in Fig 3.

## 4. Results

This section gives numerical simulations to validate the proposed formation control and trajectory tracking framework under disturbances. In this work, it was assumed that a low-level attitude controller could perfectly follow the speed commands given by the upper layer. Simulations are performed in MATLAB using a discrete-time implementation with a time step of Δt=0.01 s. A team of N=6 UAVs is considered. The total simulation time is $T_{\text{end}}=\text{1}\text{5}\text{0}$ s. The formation control gain is $k_{p}=\text{0}.\text{1}\text{5}$ and the centroid regulation gain is $k_{c}=\text{0}.\text{5}$. The desired formation offsets $f_{i}$ are $\left(\sqrt{\text{3}},\text{1}\right)$, (0,2),$\left(-\sqrt{\text{3}},\text{1}\right)$, $\left(-\sqrt{\text{3}},-\text{1}\right)$, (0,−2), $\left(\sqrt{\text{3}},-\text{1}\right)$. These define a regular hexagonal with sides 2 m long. Disturbances were incorporated into the simulations as follows: The wind is modelled as a random additive disturbance with maximum amplitude 0.04 m per time step (0.04 m/Δt). Position measurements have a random error of 0.1 m maximum. Each agent receives the UAV positions with some random delay up to 10 time steps (10Δt). Each obstacle is as a disk with radius $r_{j\;}\in [\text{0}.\text{2}\;,\text{1}]\text{ m}$. The sensing range for obstacle avoidance is $r_{\text{safe}}=\text{6}\text{ m }$with repulsion gain $k_{\text{0}}=\text{1}\text{4}$. The minimum desired inter-agent distance is $d_{\text{min}}=\text{0}.\text{5}\text{ m}$ and the safety activation threshold $d_{\text{safe}}=\text{1}\text{ m}.$ The collision avoidance gain is selected as $k_{\text{coll}}=\text{5}$.

The desired centroid trajectory is initially fixed at the origin until t=40 s, then it moves smoothy toward a target position of [20, 0] m reaching it at t=95 s, and stays at [20, 0] m until the end $T_{\text{end}}=\text{1}\text{5}\text{0}$ s. The UAVs are initialized apart from each other with the first goal being to achieve the desired formation around origin at t=40 s. The second goal is then to move to the new target position while keeping the formations. All of this must be done while avoiding obstacles and inter-UAV collisions under disturbances.

**Fig 4:**
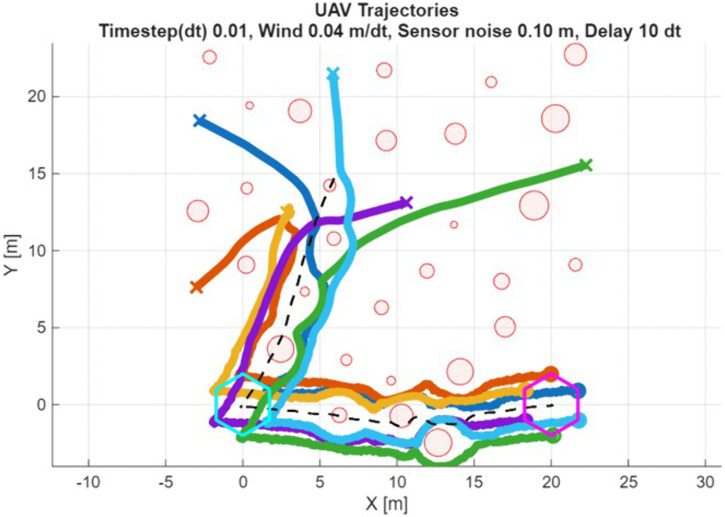
UAV trajectories for hexagon formation and tracking. Centroid trajectory (dotted), first formation location (cyan) and target location (magenta) are also shown.

Fig 4 shows the trajectories of all UAVs in addition to the centroid of the formation. In the first part the agents start from arbitrary initial positions and achieve the desired formation. In the second part, the formation is commanded to move to [20,0] m which is also performed successfully. Wind, sensor and delay type disturbances are present at all times. Static obstacles are encountered at times and on occasion the agents get too close to each other. These trigger avoidance forces which temporarily deform the shape, but the formation is recovered eventually.

**Fig 5:**
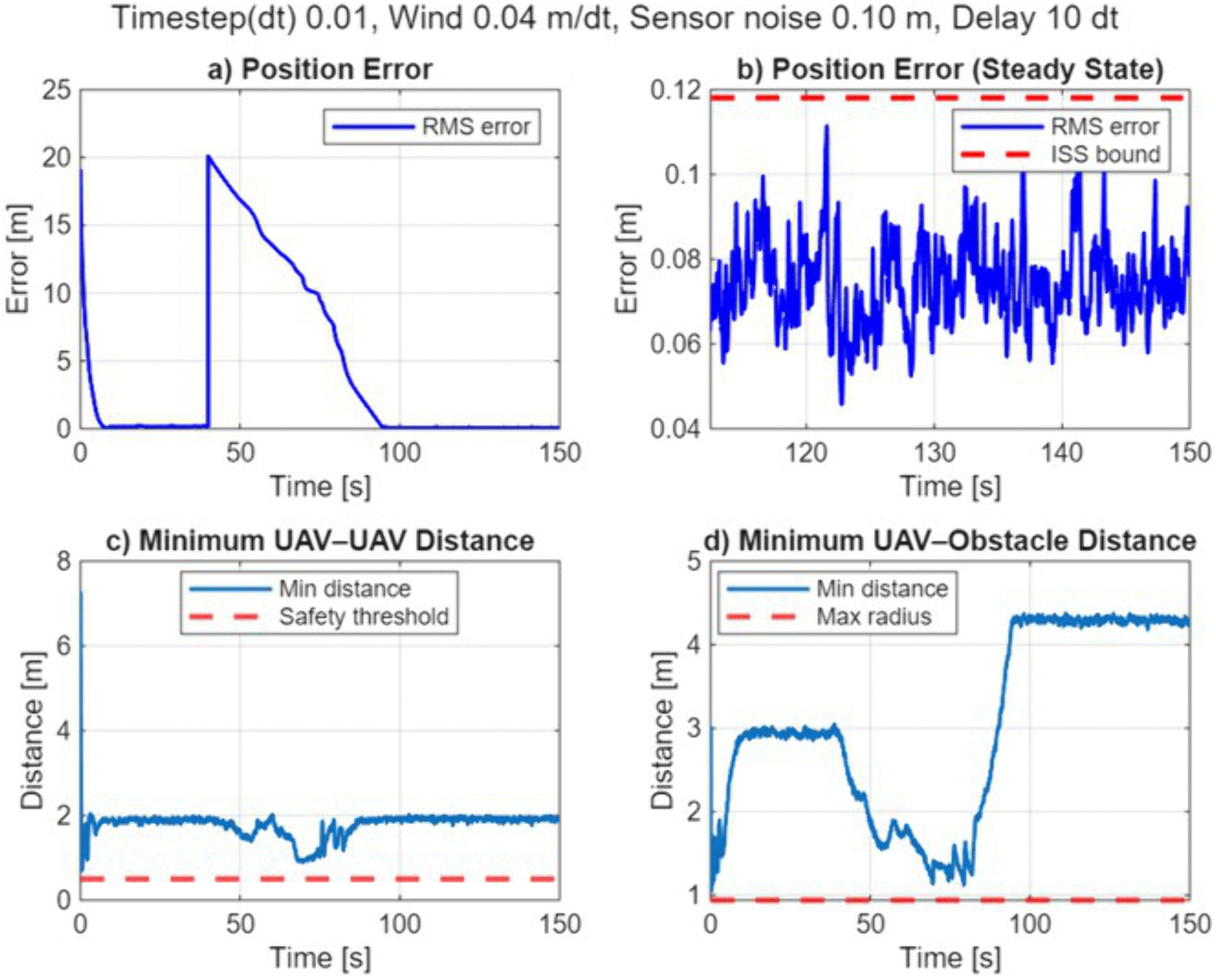
Position error and minimum distances for hexagon formation and tracking.

The formation and tracking performance are quantified using the root-mean-square (RMS) value of the errors of the 6 UAVs as shown in Fig 5-a. In the first part, i.e., 0≤t<40 s, the UAVs initialized to arbitrary locations are commanded to form a hexagon around origin and it can be seen that this achieved before t=10 s when the error becomes very small. At t=40 s the centroid of the UAV formation is commanded to move to [20,0] m so the error becomes high but then decreases towards zero before t=100 s as the formation moves to the new target. Fig 5-b zooms to the final (steady-state) portion of Fig 5-a. It can be seen that the RMS error settles within a bounded region despite the presence of wind, sensor, and delay disturbances. The dashed line is the state-state ISS bound in Theorem 2 (specifically the Remark after). In Fig. 5-b, the numerical value of the steady-state ISS bound is computed via the theoretical formula in Theorem 2. The steady-state RMS error remains below this bound consistent with the ISS framework which guarantees boundedness under bounded disturbances. Fig 5-c depicts the minimum inter-agent distance (i.e., distance between the UAVs closest to each other) over time. As predicted by the collision avoidance design, this distance remains above the prescribed safety threshold $d_{\text{min}}=\text{0}.\text{5}$, ensuring that no collisions occur even during aggressive avoidance maneuvers. Similarly, Fig 5-d shows the minimum distance between any UAV and the obstacles (i.e., distance between the UAV and the obstacle that are closest to each other) over time. The distance never falls below the obstacle safety radius $r_{\text{safe}}$, validating the effectiveness of the repulsive potential field in maintaining obstacle clearance.

**Fig 6:**
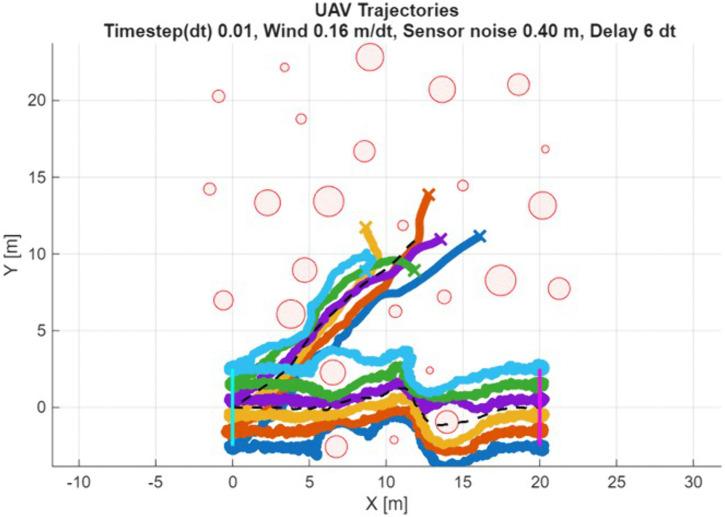
UAV trajectories for vertical line formation and tracking. Centroid trajectory (dotted), first formation location (cyan) and target location (magenta) are also shown.

**Fig 7:**
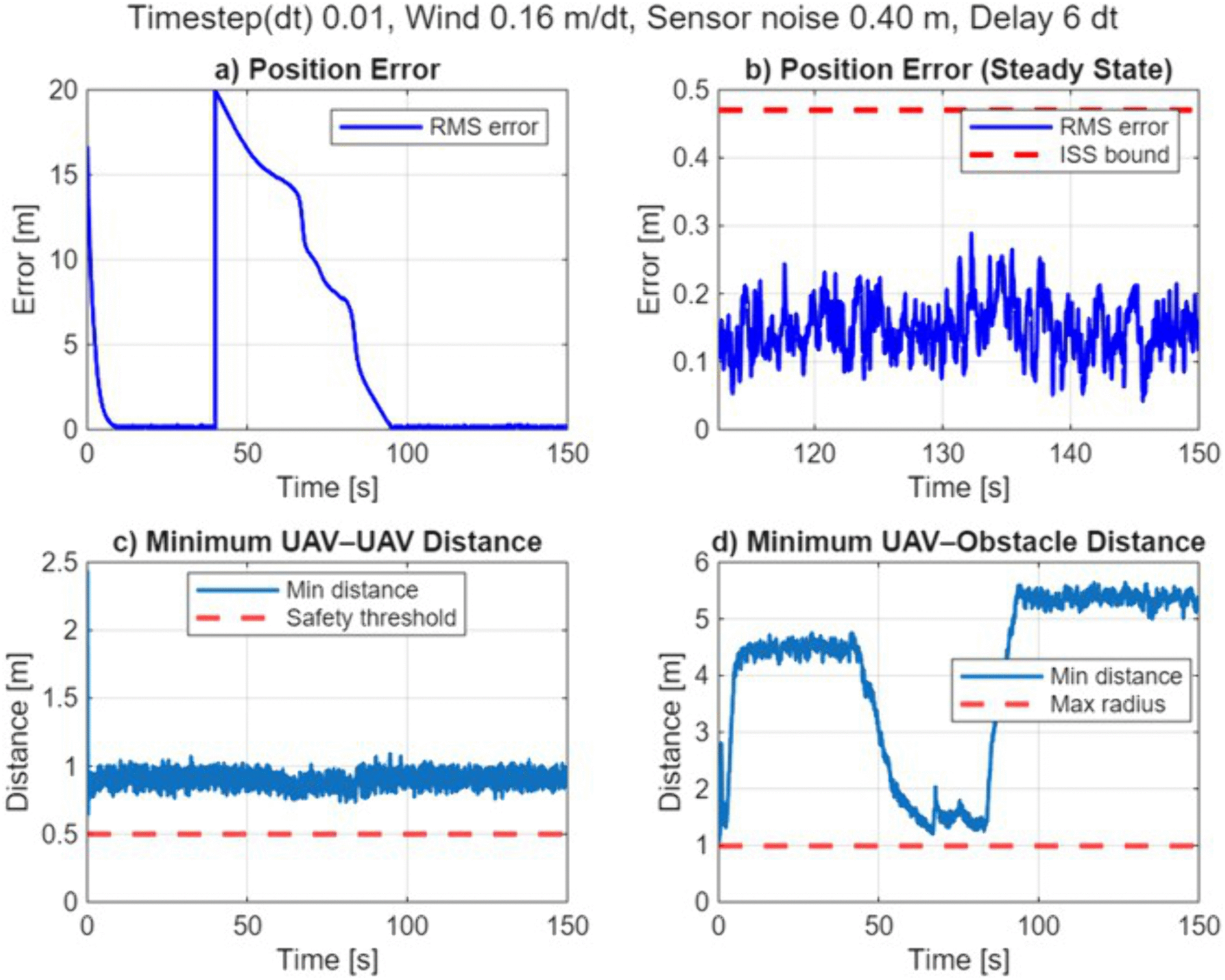
Position error and minimum distances for vertical line formation and tracking.

Figs 6 and 7 show the simulations repeated for different initial conditions, disturbance values and line formation with similar successful results. The desired formation offsets $f_{i}$ are (−2.5,0), (−1.5,0), (−0.5,0), (0.5,0), (1.5,0), (2.5,0), defining a vertical line with UAVs 1 m apart.

Overall, the results display agreement between theoretical analysis and simulation outcomes. The closed-loop system maintains formation, achieves safety with respect to both obstacles and inter-agent collisions, and accomplishes tracking performance under disturbances that is consistent with the ISS framework.

## 5. Conclusions

This paper presented a unified formation control, tracking and obstacle avoidance framework for multi-UAV systems operating under various disturbances. A consensus-based formation controller was combined with centroid tracking, obstacle avoidance, and inter-agent collision prevention. First a Lyapunov stability analysis was developed to examine the behavior for the nominal case, and global exponential stability was established for formation regulation and centroid tracking. Next, in the presence of wind, sensor and delay disturbances as well as avoidance forces, an input-to-state stability (ISS) analysis was carried out. Explicit bounds on the formation tracking error were derived which provide a quantitative link between disturbance magnitudes and steady-state performance.

Theoretical results were supported by simulation studies demonstrating reliable formation acquisition, safe navigation without inter-agent collisions, and consistent obstacle clearance for the multi-UAV framework under wind, sensor and delay disturbances. Moreover, the observed steady-state error remained within the ISS bound, confirming the earlier analysis.

Overall, the proposed framework offers a useful solution for formation control and obstacle avoidance in multi-UAV systems subject to disturbances. Future studies will focus on expanding the analysis to dynamical obstacles, incorporating actuator saturation and coupling effects, and validating the approach through experimental flight tests. Future studies are planned to focus on 3D obstacle avoidance and formation planning of swarm quadrotor UAVs.
